# Hacking Commensal Bacteria to Consolidate the Adaptive Mucosal Immune Response in the Gut–Lung Axis: Future Possibilities for SARS-CoV-2 Protection

**DOI:** 10.3390/biotech11010003

**Published:** 2022-01-30

**Authors:** Marcela Pereira, Ju Kyoung Oh, Dae-Kyung Kang, Lars Engstrand, Valerie Diane Valeriano

**Affiliations:** 1 Department of Microbiology, Tumor and Cell Biology, Karolinska Institutet, 17165 Stockholm, Sweden; marcela.pereira@ki.se (M.P.); ju.kyoung.oh@ki.se (J.K.O.); lars.engstrand@ki.se (L.E.); 2 Department of Animal Resources Science, Dankook University, Cheonan 31116, Korea; dkkang@dankook.ac.kr

**Keywords:** mucosal immunology, mucosal vaccines, mucosal pathogens, COVID-19, gut microbiome, epitope

## Abstract

Infectious diseases caused by mucosal pathogens significantly increase mortality and morbidity. Thus, the possibility to target these pathogens at their primary entry points can consolidate protective immunity. Regarding SARS-CoV-2 infection, it has been observed that the upper respiratory mucosa is highly affected and that dysregulation of resident microbiota in the gut–lung axis plays a crucial role in determining symptom severity. Thus, understanding the possibility of eliciting various mucosal and adaptive immune responses allows us to effectively design bacterial mucosal vaccine vectors. Such design requires rationally selecting resident bacterial candidates as potential host carriers, evaluating effective carrier proteins for stimulating an immune response, and combining these two to improve antigenic display and immunogenicity. This review investigated mucosal vaccine vectors from 2015 to present, where a few have started to utilize *Salmonella* and lactic acid bacteria (LAB) to display SARS-CoV-2 Spike S proteins or fragments. Although current literature is still lacking for its studies beyond in vitro or in vivo efficiency, decades of research into these vectors show promising results. Here, we discuss the mucosal immune systems focusing on the gut–lung axis microbiome and offer new insight into the potential use of alpha streptococci in the upper respiratory tract as a vaccine carrier.

## 1. Introduction

Infectious diseases caused by mucosal pathogens have significantly increased mortality and morbidity worldwide. At present, scientists are again reminded of this challenge and the requirements for vaccine development to tackle imminent threats such as the severe acute respiratory syndrome coronavirus (SARS-CoV-2). COVID-19, caused by the SARS-CoV-2 virus, has infected 219 million people and taken 4.55 million lives globally since 2019, affecting daily life. The virus also continues to evolve, and although currently licensed vaccines have some level of protection for the Alpha (B.1.1.7) and Beta (B.1.351) variants [1,2], they are unable to provide complete coverage, specifically for the emerging variants such as the Delta (or B.1.617.2) and Omicron (or B.1.1.529) variants, which have spread quickly in many countries.

The primary design strategies for SARS-CoV-2 vaccine development focused on two tracks: (1) usage of whole viruses, either as inactivated or live-attenuated vaccines (Sinovac-CoronaVac), or (2) use of genetically engineered vaccines, such as in recombinant DNA, mRNA vaccines, and viral-vector-based vaccines that utilize a different modified virus as a delivery vector. As of 1 September 2021, the FDA-authorized vaccination strategies include mRNA vaccines (Pfizer-BioNTech and Moderna) and a viral-vector-based vaccine (Johnson and Johnson/Janssen) [3]. The latter is provided as an emergency-use-only vaccine but has gained more traction as a viral-vector-based system in comparison to the Oxford-Astra Zeneca (ChAdOx1 nCoV-19). The other vaccine contender currently being developed by Novavax is the use of protein subunits, utilizing purified forms of the viral proteins rather than the attenuated virus itself.

Of the different vaccine design strategies, first- and second-generation vaccines include more traditional designs based on whole inactivated pathogens or the purified native proteins of the pathogens [4]. Nevertheless, because of the necessity for quickly developing vaccines against the COVID-19 pandemic, the next-generation vaccines seem to have taken center stage due to their possibility for development using sequence information alone [5]. Looking forward, despite the observed effectiveness of these currently rolled out vaccines, further improvement is required to effectively target the emerging variants. In this sense, third-generation vaccine development using reverse vaccinology (RV) provides avenues for potential solutions towards long-term management worldwide.

RV presents an opportunity to improve vaccine design with possible ready-made vectors, processes, and formulations. Likewise, research in this field can improve vaccine target discovery, aid functional epitope search and structural information databases for use in similar pathogens, and expand other possibilities in this field [6]. Such techniques allow the design of better multiepitope vaccines, which may have higher efficacy against old and new variants of SARS-CoV-2 and similar coronaviruses [7,8].

Such RV designs continuously evolve to enhance vaccine safety. However, RV is still reliant on adjuvants to increase the efficacy of some vaccine formulations, as it only relies on sequence-based information to predict antigenic determinants, but not immunogenicity. It may require specific sequences upstream or downstream that represent motifs recognized by the immune system. Thus, RV has evolved towards SBRV (structural-based reverse vaccinology), which greatly enhanced the immune response determination. However, the field of immunoinformatics requires much development to better understand antigenicity and immunogenicity [9]. Currently, available resource tools such as the Immune Epitope Database (IEDB) include validated methods for identifying MHC class I and class II binding. However, the immune response is more complex than antigenic binding, as it requires prediction of the antigen processing, T cell and B cell epitope recognition, followed by the type of induced immunoglobulin. To come closer to a complete prediction system, further studies and an extensive experimental dataset are needed since it will allow for the analysis of variables such as conservation of regions in complete genomes, population coverage, and pathogen-specific immunoregulation [6]. 

Similarly, a better understanding of the complex symbiotic interactions between the host and commensal bacteria in mucosal niches can be achieved from insights from the Human Microbiome Project. Elucidation of this interplay between the microbiome and specific host immunoregulation pathways is invaluable in the search for immunogenic adjuvants. It extends the target repertoire in the RV field when utilizing the commensal microbial communities as a possible inducer of innate cell-mediated immunity and cell-mediated cellular immunity through an interaction with the mucosal epithelium. Such co-existence provides protective functions and contributes to immunological tolerance, allowing the microbial gut ecosystem to reach homeostasis [10].

In this perspective, insights towards mucosal vaccination and specially designed bacterial mucosal vaccine vectors gained some attention, particularly in the possibilities of vaccine-induced microbiome alteration and the potential to manipulate and hijack the capacity of commensal bacteria in instigating specific immune responses. Thus, this review aims to summarize and discuss recent findings that correlate mucosal immunity to the microbiome and the tools that microbes use to interact with the host immune system. The review’s focus will mainly be on topics relevant to the gut–lung axis. Through this, we also search for the windows of opportunity towards the development of mucosal vaccine designs based on bacterial vaccine vectors that could be effective in infections such as the one caused by SARS-CoV-2.

## 2. Literature Search Method

In this review, we aimed to find manuscripts published between 2005 and 2021. The search was divided into separate search terms grouped as follows: “epitope [Title/Abstract] AND Mucosal Vaccines OR Mucosal Vaccine [Title/Abstract] AND human [Title/Abstract]”, “Microbiome [Title/Abstract] AND Mucosal Immunity [Title]”, “Microbiome [Title/Abstract] AND Mucosal Vaccines OR Mucosal Vaccine [Title/Abstract] AND human [Title/Abstract]”, “microbiome [Title] AND Mucosal Vaccines OR Mucosal Vaccine [Title] AND Sars-Cov-2 OR COVID-19 [Title]”, “Pyrophosphates OR quorum-sensing molecules OR bacterial cyclic-di-GMP OR bacterial cyclic-di-AMP OR flagellin OR Muramyl dipeptide [Title] Immune activation [Title/Abstract]”. All searches were performed on PubMed (Bethesda, MD, USA), and the last search was performed on 10 October 2021. Papers were mostly curated focusing on third-generation engineered bacterial vectors being tested as mucosal vaccines relevant to the gut–lung axis, either via surface display or secretion mechanisms, and their potential role in microbiome dynamics and mucosal immunity. Papers listed in the results from the search terms but not directly related to the review topic were omitted.

## 3. Application of Reverse Vaccinology in Bacterial Mucosal Vaccine Vector Designs

Epitope discovery and synthetic vaccine designs have now gained traction thanks to the help of immunoinformatics. RV and SBRV are based on the principle that protective monoclonal antibodies (mAb) are raised towards target pathogenic epitopes, with the assumption that it will produce an immunogenic polyclonal antibody (Ab) response similar to the protective mAb, when used as an immunogen [7,9,11].

However, the immune response elicited by an immunogen is distinctly more complex due to the presence of different antigen-binding regions (ABRs), which account for discontinuous epitopes, known as “paratopes”, that make up various regions of the protein antigen [11,12,13]. Such difference between the elicited immune responses led to the failure of most predicted B-cell synthetic linear peptide vaccines, but also provided the knowledge needed for the improvement of future endeavors, as experimental approaches have shown that targeted immunogenic domains can exceed the effective protection provided by the whole purified protein [2]. Considering this, it is important to combine both RV and SBRV to provide the most accurate prediction of the amino acid sequence of the discontinuous epitopes that comprise most B cell epitopes [9].

Another relevant issue that needs to be addressed in the development of RVs is that in many cases, these peptide antigens induce lower levels of immune response. It would require an immunogenic structure such as liposomes [14], outer membrane vesicles [15], inorganic or organic adjuvants [16], or carrier proteins. Examples of such structures are the recombinant Hepatitis B Core Antigen (HBcAg) protein [17], and the Freund’s adjuvant (considered a “gold standard”), water-in-oil emulsion of heat-killed mycobacteria [18]. Multiple other attenuated or heat-killed bacteria have also been tested as adjuvants, including bacterial alternatives generally regarded as safe (GRAS), such as lactic acid bacteria (LAB), and potentially other commensal microbes.

**Figure 1:**
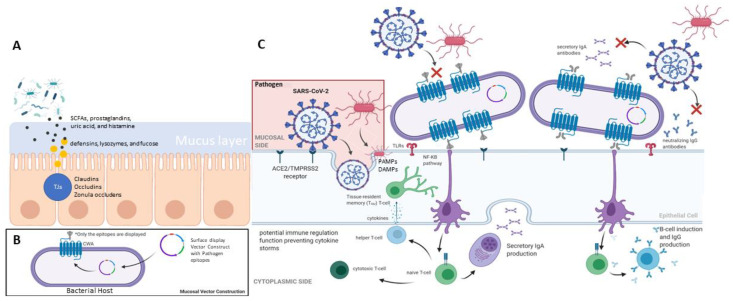
Summary of mucosal-associated immunity in human (**A**) gut barrier system, (**B**) mucosal vector construction, and (**C**) the mucosal response elicited by immune cells. (Adapted from “Remdesivir” [19]).

We discuss more about the recent developments of various bacteria used as RV vector platforms in Section 5. Given the ongoing pandemic, many research groups have designed and tested such RV platform designs to display predicted antigenic epitopes from the Spike S protein of SARS-CoV-2 on LAB (see Figure 1). Small regions such as the expression of the receptor binding domain are possible to elicit an IgA response, even without the addition of other adjuvants in either *Lactobacillus plantarum* or *Mycobacterium paragordonae*. Nevertheless, for a better understanding of this intricate balance and the ability to stimulate an immune response, it is necessary to get a better overview of the ecosystem within specific host mucosa-associated niches such as the oral, upper respiratory tract, gut, and cervicovaginal mucosae, considered to be the hotspots for these bacteria–bacteria and bacterial–host immune cell interactions.

## 4. Microbiome and Mucosal Immunity

### 4.1. Overview of the Mucosal Immune System

The mucous membrane, the mucosal immune system, and the mucosal-associated lymphoid tissue (MALT) are the first lines of defense of the body against foreign matter. It covers the digestive, respiratory, urinary, and genital tracts, the eye conjunctiva, exocrine gland ducts, and inner ear. In sum, the body surfaces in contact with the exterior and not covered by skin are 200× bigger than the skin’s total coverage area [20]. The main functions of the mucosal immune system can be divided into three: (1) to protect the body against potentially harmful microorganisms, (2) to prevent the uptake of undegraded antigens derived from food, air, and commensal microorganisms, and (3) to avoid the development of allergic response in case the antigens as mentioned earlier get into the body [21,22].

Although they have many similarities, it is essential to notice that choosing the correct MALT subdivision is crucial to achieving the desired immunization after vaccination. The oral immunization will produce antibodies in sites such as the intestine and mammary and salivary glands, while nasal or tonsillar immunization will evoke responses in the upper airway mucosa (saliva, nasal secretions) and cervicovaginal mucosae [21].

Here, we aim to focus on three of the subdivisions of the MALT, the gut-associated lymphoid tissue (GALT), the nasopharynx-associated lymphoid tissue (NALT), and the bronchus-associated lymphoid tissue (BALT) and their crosstalk with the microbiome. Reviews on other body sites [23,24,25,26], and the development and differentiation of GALT, NALT, and BALT, are out of the scope of the present review but can be found elsewhere [21,27].

#### 4.1.1. GALT

Briefly, this body site where mucosal-associated immunity happens is characterized by mucosal-associated lymphoid tissue in the proximity of the lumen, cells that produce a mucous layer, a single epithelial barrier making the interface between the body and the exterior environment, and activated immune cells [28]. Therefore, protection conferred by the mucosal immune-associated tissue is carried out by two different systems: physical and chemical. The non-specific physical mechanism comprises the epithelial barrier covered with a mucus layer and antimicrobial peptides. Among the peptides present in this layer are defensins, lysozymes, and fucose. Between the epithelial cells, the tight junctions (TJs) control the movement of soluble molecules between environments. TJs comprise junctional adhesion molecules, claudins, occludins, and zonula occludens proteins. Some microbial metabolites can modulate the expression of TJs, for instance, SCFAs, prostaglandins, uric acid, and histamine [21,28,29].

The mucosal response elicited by immune cells in this tissue is highly dependent on the type of antigen encountered. This response is triggered when an antigen comes in contact with the intestinal epithelial cells (IEPs). For instance, non-pathogenic antigens will elicit various regulatory T cell types of responses. In contrast, the presentation of pathogenic antigens will trigger ‘danger signals’, activating mucosal antigen-presenting cells (APCs, such as dendritic cells (DCs), B lymphocytes, and macrophages) through pattern-recognition receptors (PRRs) in response to the presence of pathogen-associated molecular patterns (PAMPs) or danger-associated molecular patterns (DAMPs) and increased levels of Th1 and Th17, among other pro-inflammatory immune cell profiles [21,22,30].

#### 4.1.2. Inductive Sites and Immune Cell Response to Pathogens

In the GALT, exogenous stimuli come directly from the mucosal surfaces via M-cells, probably aided by dendritic cells (DCs) [20]. These DCs present in the gut are CD103+ and express CCR9 and α4β7 by responding to T and B cells through a retinoic acid receptor-dependent mechanism [30].

When immature DCs have sampled pathogenic bacteria from the lumen, it will trigger a process of accumulation of new DCs, which will increase the bacteria’s engulfment rate. This increase in effector DCs will increase Th1 and Th17 effector cells, which are CD4+ T helper cells that favor a pro-inflammatory microenvironment. 

To modulate this inflammatory state, immature DCs are stimulated to become tolerogenic DCs (tolDCs). TolDCs will then induce the differentiation of naïve CD4+ T cells into regulatory T helper cells (Treg), which will suppress mucosal inflammation through deactivation of effector dendritic cells and suppression of Th1 responses, and they will also cause a shift of Th17 into Treg subsets. This mechanism is also present in the so-called “oral tolerance”. In “oral tolerance”, the stimulation of T cells into a Treg phenotype is performed by mucosal dendritic cells (DCs), which carry the microbial and dietary antigens to the mesenteric lymph and present it to T cells [20]. When repeated activation of DCs by the same innocuous antigen occurs, DCs will no longer activate an inflammatory state, which characterizes a state of tolerance.

Another subset of T cells that plays a crucial role in mucosal immunity is the gamma delta T cells (γδ T-cell). Gamma delta T cells are among the most numerous “antigen-specific” T cell subsets in peripheral blood and mucosal tissues, including the lung and intestine. These T cells present three distinct functions: detect common antigen or host molecule generated by microbial infection, stress, and/or malignant transformation [31], and their δ-chain will vary according to function [32]. These cells are not dependent on major histocompatibility complex (MHC) but require CD3 complex proteins for function and have cytotoxic effector activity [32]. Moreover, they can also activate antigen-presenting cells and/or direct stimulation of other mucosal leukocytes [31].

Dendritic cells are also pivotal to the activation of B cells, and such activation can be T cell-dependent or independent. Several factors are related to the activation of B cells to produce IgA. The literature shows that retinoic acid (RA) derived from vitamin A plays a crucial role in the differentiation of naïve B cells into an IgA-producing phenotype with expression of α4β7 and the CC chemokine receptor CCR9. Further activation of PRRs by PAMPs and DAMPs shapes APCs’ phenotype in GALT, promoting the expression of retinaldehyde dehydrogenase (RALDH) and inducible nitric oxide synthase (iNOS). Increased levels of nitric oxide (NO), in turn, lead to the release of the innate switch factors APRIL (A PRoliferation-Inducing Ligand) and BAFF (BlyS), which in the presence of IL-10 and IL-4 trigger the switch of B cells to an IgA-producing phenotype [21].

One of the key players in mucosal immunity is secreted IgA (SIgA), which comprises 80% of the total secreted antibodies in an adult. SIgA promotes immune exclusion by coating microorganisms, limiting epithelial contact and penetration. The SIgA is secreted by pIgR-mediated export, and it is fundamental to keep the body’s homeostasis. This is proven by literature reports that show mortality rates in infants in correlation to breastfeeding since it provides SIgA from the mother to the child and prevents infections in the gastrointestinal and respiratory tracts [20,33]. SIgA cannot kill microorganisms because it does not have bacteriolytic or complement activation effects, but it inhibits adherence of microorganisms to the epithelial cells by neutralizing and agglutinating activity [34]. In opposition, when IgG binds to the bacteria, the bacteria will be destroyed through lysis and complement activation. In this sense, it is more desirable to promote a B switch into IgG than IgA in the context of mucosal immunization, and such switch is T cell- and toll-like receptor-4-dependent. Still, the entire activation mechanism of the IgG isotype is not as well-known as the one for IgA [35].

#### 4.1.3. Activation of Immune Response by PAMPs and DAMPs

In the presence of pathogen-associated molecular patterns (PAMPs) or danger-associated molecular patterns (DAMPs), the immune system is activated by activation of the germline-encoded PRRs. PRRs are a group of receptors that include Toll-like receptors, RIG-I-like receptors, NOD-like receptors, C-type lectin receptors, retinoic acid-inducible gene I-like receptors (RLRs), and nucleotide-binding oligomerization domain-like receptors (NLRs) [36]. Once activated, these receptors will trigger the production of type I and III IFNs, pro-inflammatory cytokines, and chemokines.

DAMPs are cellular components released after injury in the tissue, caused, for example, by some pathogen. They include RNA, DNA, heat-shock proteins (HSPs), high-mobility group box 1 (HMGB1), proteoglycans, fibrinogen, fibronectin, S100 proteins, and purine metabolites [37]. PAMPs (also called MAMPs—microbe-associated molecular patterns) are components of the microorganisms that can elicit an immune response. Considering the focus of this review, we will focus on the PAMPs, more specifically, the vita-PAMPS, considering that the literature shows that the immune system preferentially targets live microorganisms [38,39], and that they are the ones used in mucosal vaccines.

#### 4.1.4. Vita-PAMPs

Studies have shown the capacity of the immune system to differentiate between live and dead microorganisms. For instance, a strong immune response activator is mRNA, which rapidly degrades once the microorganism dies [38,39]. Metabolites also seem to be capable of acting as vita-PAMPs. The literature cites bacterial pyrophosphates [31,40], quorum-sensing molecules [41,42,43,44,45], bacterial second messengers such as cyclic-di-GMP [46,47] and cyclic-di-AMP [46,48,49,50], LPS [51,52], and flagellin [53] as vita-PAMPs.

Pyrophosphates are derived from the bacterial non-mevalonate pathway of isoprenoid biosynthesis (‘phosphoantigens’—(E)-4-hydroxy-3-methyl-but-2-enyl pyrophosphate (HMB-PP)) and expressed in several pathogenic bacteria. They activate γδ T cells and induce expression of pro-inflammatory markers such as tumor necrosis factor-α and interferon-γ [31,40].

Quorum-sensing (QS) molecules differ depending on the Gram status of the bacteria. Acyl-homoserine lactones (AHL)-based QS is present in Gram-negative bacteria, oligopeptide-based QS in Gram-positive, 4,5-dihydroxy-2,3-pentanedione (DPD, AI-2)-based QS between Gram-positive and -negative, and the quinolone-based QS in certain bacteria, including species of the genera *Alteromonas*, *Burkholderia,* and *Pseudomonas* [45]. QS signaling molecules are crucial to producing virulence factors and biofilm formation [41] and activate the immune system. According to Zimmermann and co-workers [41], AHL can promote chemotaxis and recruit polymorphonuclear neutrophils (PMNs) to the local biofilm formation. Kim and co-workers reported TLR4 activation in the presence of QS molecules. However, studies show that they may have a pro-inflammatory (activation of IL-1, IL-6, IL-8, Cox-2, PGE2) and anti-inflammatory (activation of IL-10 and attenuation of the immune response against other PAMPs such as LPS) factor, which allow the bacteria to produce QS molecules to evade the immune response [41,42,43,44].

Dinucleotides are a good target for the immune system activation because cyclic dinucleotides (made of two nucleotides linked by two phosphodiester bonds) such as cyclic-di-GMP (cGMP) and cyclic-di-AMP (cAMP) can vary in the position of their phosphodiester bonds, and bacteria present a specific configuration different from the one found in eukaryotes [47,54]. The cGMP is a ubiquitous second messenger present in a range of bacteria, related to biofilm production, cell–cell signaling, motility, and expression of virulence [47,48], while the cAMP is important to bacteria growth, osmotic control, and sporulation [54]. To date, the literature reports three distinct receptors capable of identifying cyclic dinucleotides and activating immune response: STING, DDX41, and RECON. STING (STING-TBK1-IRF3 signaling pathway) and DDX41 (binds to cyclic dinucleotides and enhances STING affinity) leads to the activation of type I interferons and cytokines, leading to pathogen elimination, and RECON (or AKR1C13) binds specifically to cAMP and increases the levels of NF-kB (nuclear factor kappa-light-chain-enhancer of activated B cells) [47,54,55].

LPS, a major component of the cell membrane in Gram-negative bacteria, is a macromolecular glycolipid composed of a hydrophobic lipid A region and a long-branched carbohydrate chain. It initiates a potent immune response that is TLR4-dependent. After binding to the complex TLR4-MD-2 (LR4 (mTLR4)/myeloid differentiation factor 2 (MD-2)), it will trigger the production of NF-κB, IRF3, and pro-inflammatory cytokines [51,52].

Finally, flagellin is a structural protein part of the flagellum, and it is required for bacterial motility and is also related to adhesion and invasion. Flagellin can activate the TLR5, and activate the MyD88 cascade and MAPK pathways [53].

All the above-mentioned PAMPs and vita-PAMPs are candidate targets for developing new bacterial-based mucosal vaccines (summarized in Table 1). However, more importantly, the choice of the bacteria is fundamental since adding the specific PAMP to another species seems not to produce the desired output [55,56]. For example, Yang and co-workers [56] observed that the presence of filamentous bacteria (SBF) could induce the production of Th17 via antigen derived from it, but if the same antigen is expressed in *Listeria monocytogenes,* Th1 is produced instead.

### 4.2. NALT and BALT

The upper airway is exposed to many pathogens daily, and to avoid infection and further complications in the upper and lower respiratory tract, it has several defense mechanisms. The first layer of defense in the upper respiratory tract presents high levels of SIgA (similar to the GIT), mucociliary transport, and secretion of bactericidal enzymes (similar to the lower respiratory tract). Combining these systems, the SIgA can bind the bacteria, and the mucociliary system excretes it. IgG also plays a role in this tissue, and it is responsible for lysing the bacteria and complement activation [34]. The second nasal immunological barrier is composed of a network similar to the one found in the GIT, presenting microfold M cells, macrophages, innate lymphoid cells, dendritic cells, and B and T lymphocytes [34,69]. Interestingly, the presence of bronchus-associated lymphoid tissue (and IgA-producing mucosal B cells) is more common in children and adolescents than adults, and that could be correlated to the fact that younger individuals present a milder case when infected with SARS-CoV-2 in comparison to older adults [70,71], showing how local immunization may be a good option to avoid the severity of COVID-19 cases.

Unlike the GIT and nasal cavity, the lung is considered a sterile organ. To allow for gas exchange, the membrane present in the lungs must be extremely thin. It is only 1–2 µm thick (roughly 1/10th the diameter of a cell nucleus). This would be an easy target for pathogens. To defend this body site, the lung is equipped with highly specialized mechanisms of defense, such as specialized cells of the immune system, including alveolar macrophages and dendritic cells, a mucus barrier, ciliated epithelial cells (types I and II), non-ciliated mucous goblet cells, club cells, and undifferentiated basal cells [72]. According to Weitnauer and co-workers [72], it is believed that the lungs are maintained in a “hyporesponsive” state to avoid chronic inflammation; in this state, the mucociliary clearance system (MCC) and macrophages present in the lumen would be responsible for the clearance of dead bacteria and other pathogens that reach this area. There are two main theories of what would cause the switch between an anti- and pro-inflammatory state for this specific tissue: the load threshold theory and the viability-associated (vita)-PAMPs. In the threshold theory, the activation of macrophages and production of pro-inflammatory cytokines would be triggered by the amount of antigen inhaled, exceeding the capacity of clearance by MCC and lumen macrophages [73]. While in the vita-PAMPs theory, only markers that would present in viable bacteria would elicit a pro-inflammatory response of the lung [39].

In this sense, any colonizing species naturally present in the upper respiratory tract could be targeted to produce mucosal vaccines for the respiratory tract since it will elicit a lung response if it exceeds the average load observed there [34,73].

## 5. Bacterial Mucosal Vaccines in the Literature

### 5.1. Bacterial Vector Selection and Genetic Engineering for Surface Display of Antigens

Traditionally, in second-generation vaccine technology, *E. coli* was used as a recombinant expressing antigen that was later purified and used as a protein subunit vaccine [73]. Seeing the potential as adjuvants or as tolerogenic stimulants and the type of immune response triggered by various bacteria, several bacterial shuttle vector systems have then been tested for use as gene delivery systems. In this system, the bacteria were no longer only used to produce a protein that will be further purified to produce a vaccine, but the bacteria will be used as the carrier. Here, we will be focusing primarily on the use of engineered live bacteria as mucosal vectors, while the use of bacteria as adjuvants can be found elsewhere [19,74].

In principle, the bacterial cell-surface display allows either proteins and/or peptides to be presented on the surface of microbial cells using anchoring motifs as fusion proteins. There are many ways to display them, as described by Lee and co-workers [75]. Surface-displayed peptides or proteins are passengers on carrier proteins that are either terminally fused to the N- or C-regions or use a sandwich fusion where the peptide is inserted between the carrier protein. The choice of carrier proteins is usually based on those exposed to the bacteria’s outer regions, collectively described as surface proteins. These include a covalently anchored outer membrane or cell wall proteins such as SrtA (sortase)-dependent LpXTG-anchored proteins, lipoproteins, and other N- or C-terminally anchored proteins that undergo a Sec-dependent translocation pathway. Other non-covalently anchored proteins include LysM domain-containing proteins, choline-binding domain-containing proteins, peptidoglycan domain-containing proteins, S-layer proteins, or WxL domain-containing proteins. For vaccine antigen display, these surface proteins are primarily relevant to stimulation of the immune response, inclusive for these reasons of flagellar and fimbrial proteins, as mentioned in the previous section regarding potential immune-activating molecular fragments. From 2015 to the present, bacteria used as mucosal vectors include *Salmonella* spp., *Listeria* spp., *Bacillus* spp., *Lactococcus lactis*, *Bifidobacterium* spp., *Lactobacillus* spp., and other novel bacterial vectors (summarized in Table 2).

In recent years, various gastrointestinal disease-causing bacteria have been tested as live-attenuated mucosal-delivery vaccine systems. These include *Salmonella Typhimurium* (*S. Typhi*) due to its known pathogenicity mechanisms that allow it to hijack and infiltrate immune cells [94,95]. Several clinical trials have been performed in the past three decades of development [96,97], and *S. Typhi* has been continuously used in experimental designs for vaccines against respiratory infections. For example, Juárez-Rodríguez and co-workers [76] constructed a translational fusion for the synthesis of two copies of ESAT-6 (early secreted antigenic target 6 kDa) plus CFP-10 (culture filtrate protein 10) fused to the OmpC (outer membrane porin C) signal sequence of live-attenuated *Salmonella*. Such construct’s efficacy was evaluated using an aerosol challenge of *M. tuberculosis* in mice. On the other hand, Bumann and colleagues [77] developed the live-attenuated *S. Typhi* (strains CVD908 and Ty21a), displaying a recombinant fusion protein containing the OprF and OprI (which are highly conserved outer membrane proteins from the porin family present in *P. aeruginosa*) as an antigen. Their results showed an increase in serum antibody titers for IgA and IgG in volunteers. Furthermore, Frey and colleagues [78] showed a strong IgG and IgA response in volunteers using the live-attenuated *Salmonella* vaccine expressing the surface protein antigenic A (PspA) against *Streptococcus pneumoniae*. They observed that *S. Typhi* ISP1820 and *S. Typhi* Ty2 RpoS+ (RNA polymerase, sigma S, also called katF) elicited IgA anti-OMPs, IgA and IgG anti-LPS, and IgA anti-PspA in some volunteers. They also corroborate previous studies showing that RpoS+ showed higher immunity than RpoS– strains. Lastly, Piao and colleagues [79] suggested a Cytolysin A (ClyA) delivery system containing the Spike protein from SARS-CoV1 (S1E) in attenuated *S.* *Typhimurium* as a new live vaccine candidate and observed the presence of the anti-S1E antibody in the mouse serum [79].

Another pathogen, *Listeria monocytogenes*, has also been consistently used in its live-attenuated form of vaccine. Jia and colleagues [80] showed the usage of attenuated Listeria-vectored vaccines to express the *Mycobacterium tuberculosis* 30 kDa major secretory protein (r30/antigen 85B (Ag85B)) (rLm30) to boost BCG vaccinations against tuberculosis based on *Mycobacterium bovis*. In their comparative studies, rlmaIII/a30 showed significantly enhanced protection as compared to only BCG vaccination in mice, inducing strong antigen-specific T cell responses, such as splenic and lung CD4+ T cells increased expression of interferon gamma (IFN-γ), tumor necrosis factor alpha (TNF-α), and interleukin-2 (IL-2), and CD8+ T cells increased expression of IFN-γ [80].

*Listeria* and *Salmonella* have been widely used as vaccine vectors in different diseases, including cancer immunotherapy [98]. However, it is crucial to keep in mind the safety issues regarding the use of live-attenuated pathogenic bacteria [99] as a recombinant delivery system due to their potential for reversion to the pathogenic state, which requires further studies. Therefore, commensal bacteria have been studied to increase the possibility of safety without losing efficacy or changing the stimulated immune response.

Some of these commensal bacteria tested include *Bacillus* spores, such as *Bacillus subtilis* (*B. subtilis*), which have been used as a live vaccine vector system due to their safety, vitality, secretion ability, and probiotic characteristics. *B. subtilis* have been used in various studies as a vaccine carrier for viruses, pathogenic bacteria, and parasites in animal models [100]. In addition, Oh and co-workers [81] used *B. subtilis* spores to express the protective antigen (PA) from *Bacillus anthracis* and tested the efficacy of the construct to elicit an immune response using several administration routes in mice. Their studies observed that independent of the administration route, the mice presented increased levels of active antibody titer, isotype profiles, toxin-neutralizing antibody in sera, and IgA in saliva after treatment.

Sibley and colleagues [82] expressed the tuberculosis antigen MPT64 in the *B. subtilis* HU58 spore and observed a Th1 response in mice models. At the same time, Das and colleagues [83] used the *B. subtilis* PY79 strain to express a truncated fusion of Ag85B and CFP10 antigens (T85BCFP) in their spores (MTAG1 strain) or cytosol (MTAG 2 and MTAG 3 strains), and observed increased serum IgG levels and IFN-γ-producing cells in the spleen of mice.

Nevertheless, the development of oral mucosal vaccine delivery platforms also considers the amount of antigen that is still being expressed after it passes through the host digestion system. In this regard, *Lactococcus lactis* (*L. lactis*), a commonly used starter for fermented foods such as cheese and yogurt, is a promising candidate. Oral administration of acid- and bile-resistant *L. lactis* recombinant strain MG1363/pSECN to mice has demonstrated exciting results [83]. Pei and co-workers [84] observed a significantly higher antibody induction for the nucleocapsid protein of SARS-CoV-1 using an engineered *L. lactis* strain secreting the N-protein compared to the purified recombinant N-protein from *E. coli*. Furthermore, Medina and colleagues [101] reviewed *L. lactis* as a delivery vector for pneumococcal respiratory infections, and their review reports the use of proteins (PspA, PsaA, PppA, PpmA, SlrA, IgA1p) and polysaccharides (serotype 3 and 14) from *S. penumoniae* strains as antigens. *L. lactis* is widely used as an industrial LAB for heterologous protein expression. Still, some strains only survive for a few hours in the GIT, compared to some lactobacilli that show survival times greater than seven days [102].

Thus, another commensal candidate being developed with high tolerance to acid and bile stress is lactobacilli. Lactobacilli have been recently suggested as a potential adjuvant for several vaccine designs, demonstrating modulation of both innate and adaptive immunity in clinical studies, especially in gastroenterological diseases such as rotavirus, cholera, and *Salmonella* infection [103], and additionally for respiratory infections such as Influenza, SARS-CoV-1, pneumonia, and *Bacillus anthracis*, to mention a few [104].

Oliveira and colleagues [85] observed an increase in serum levels of IgG and mucosal levels of IgA in the respiratory tract of mice after treatment with four distinct LAB strains (*L lactis*, *L. casei*, *L. plantarum*, and *L. helveticus*) expressing the pneumococcal surface adhesin A (PsaA) antigen from *S. pneumoniae.* The authors mention that the capacity of the lactobacilli to persist in the mucosa plays an essential role in the strength of the elicited immune response, and therefore, certain *Lactobacillus* strains have intrinsic properties that make them more suitable for vaccine applications. For instance, their results have demonstrated that *L. plantarum* and *L. helveticus* elicited a more robust response. Still on the topic of respiratory tract infections, Chowdhury, Li, and colleagues [86,87] aimed to develop a vaccine against the influenza virus. Their team engineered *L. casei* to display influenza antigens such as consensus matrix protein-2 (sM2) and hemagglutinin (HA1) conjugated with cholera toxin subunit A1(CTA1). The constructs increased IgG and IgA levels and could protect against divergent influenza types in the challenge assay performed in BALB/c mice [86,87]. Lastly, Lee et al. [88] evaluated a vector system for SARS-CoV-1 therapeutics in a mouse model. They selected and reported which segment of the S protein from the SARS-CoV-1 virus genome was capable of eliciting an immune response after mucosal immunization.

The use of genetically modified organisms (GMOs) has been controversial over the years due to the potential of genetic instability causing unintended functionalities and the introduction of new functionalities to new bacteria in the ecosystem through horizontal gene transfer and acquisition of extracellular DNA. However, given the recent COVID-19 pandemic, opportunities with the use of genetically engineered vaccines have received a certain level of acceptance. In this case, even though Gram-positive bacteria are challenging to engineer, lactobacilli have been seen as a potential candidate to carry viral mRNA sequences. A careful design must be taken into consideration, where the selection markers for constructing vectors should not rely on antibiotic resistance selection for successful cloning, but on other selective marker genes, such as auxotrophic mutant complementation or use of the CRISPR/Cas system [105]. Once achieved, lactobacilli show promising potential not only in health promotion, immunomodulation, and tolerance [104,106], but also in its observed genetic stability after genetic manipulation [107].

The choice of anchoring strategy also requires careful consideration when developing bacterial mucosal vaccine vectors, as demonstrated by Kuczkowska and colleagues [89]. Their study evaluated the use of two distinct forms of anchoring for their fusion construct called AgE6 (it comprised Ag85B and ESAT-6 proteins from *Mycobacterium tuberculosis*) in a *L. plantarum* carrier. Although both forms could elicit a response in lymphocytes purified from TB-positive donors, and in mouse models after nasal and oral administration, they observed that the in vivo response was distinct based on the anchoring strategy.

Finally, another well-known beneficial commensal bacteria are bifidobacteria, which are established in the healthy infant’s gut microbiome. Bifidobacteria have probiotic properties and can enhance the immune response against several pathogens. Álvarez-Martín and colleagues [108] have improved the pBC1 vector systems for *Bifidobacterium catenulatum*: their construct shows good segregation stability and about 95% of retention up to 80 to 100 generations without selection. However, compared to lactobacilli, the handling of bifidobacteria presents a significant hurdle during manufacturing, as it is sensitive to oxygen. Therefore, fewer efforts have been made to use this genus as a mucosal vaccine carrier.

### 5.2. The Gut–Lung Axis: Where Is the Place of Mucosal Vaccine Vectors?

The microbiome of the upper respiratory tract (URT) performs a significant gatekeeping function in the immune barrier system [109,110,111]. Microbiome studies in mice have shown specific protection against respiratory infection via granulocyte-macrophage colony-stimulating factor (GM-CSF) signaling as one pathway [112]. Therefore, although oral vaccination may provide some form of systemic adaptive immunity, direct interference in the upper respiratory mucosal regions may be another important strategy to consider, especially when taking into consideration the previously mentioned immunization and antibody-producing sites. As said previously, nasal but not oral or intestinal immunization will evoke responses in the upper airway mucosa (saliva, nasal secretions) and cervicovaginal mucosae [21].

When targeting the respiratory tract, attention should be paid to streptococci, a promising candidate for upper respiratory tract mucosal vector vaccine development that is not yet well-studied. A recent report on the use of *Streptococcus salivarius* as a prophylactic species comes from Marchisio and co-workers [113]. Their study used the strain 24SMB, which produces bacteriocin-like substances that are active against otopathogens related to acute otitis media (AOM) events. According to their study, recolonization of the α-streptococci in the nasopharynx is correlated to a decrease of otopathogens and AOM events, showing an interesting role of this bacteria on the nasopharynx microbial ecology. Another report on the use of streptococci as a vector was presented by Shekhar and co-workers [114] using intranasal administration of *Streptococcus mitis* (*S. mitis*) to treat pneumococcal lung infection in a mouse model. In this study, they were interested in testing the protective effect of wild-type *S. mitis* and a mutant variant of *S. mitis* expressing the *Streptococcus pneumoniae* type 4 capsule (*S. mitis*-TIGR4cps) against two *S. pneumoniae* strains (D39—serotype 2, and TIGR4—serotype 4). The results obtained with the wild-type strain showed that treatment with *S. mitis* conferred immunity against *S. pneumoniae*, with higher levels of serum IgG and IgA antibodies and IL-17A independent of the strain tested. In comparison, the treatment with *S. mitis*-TIGR4cps produced a more targeted response against serotype 4. These results show the potential use of streptococci for targeted immunity against specific pathogens using genetically engineered strains. 

Moreover, the gut–lung axis has recently been recognized with relevance to the severity of COVID-19 symptoms [111]. Due to the recent SARS-CoV-1 pandemic, researchers then used similar approaches to that in examples of *Salmonella*- [79] and *Lactobacillus*-based [88] vectors expressing SARS-CoV-1 Spike S protein. For example, Wang et al. [90] expressed the whole SARS-CoV-2 S protein on the surface of the *L. plantarum* strain Lp18, and reported high production levels of the S protein with 50 ng/mL of SppIP and 0.2% bile salt for 8 h at 30 °C. Additionally, Li and colleagues [91] engineered a recombinant *L. plantarum* (LP18:RBD) expressing the receptor-binding domain (RBD) of the SARS-CoV-2 Spike protein and successfully elicited mucosal IgA in the respiratory and intestinal tract, and CD3+/CD4+ T cells in the spleen of mice. Jia and co-workers [92] also expressed Spike, envelope, membrane, and nucleocapsid proteins of SARS-CoV-2 using a live multi-deletional attenuated *Francisella tularensis* subsp. *holarctica* vector (LVS ΔcapB). Their data showed that the co-expressing membrane and nucleocapsid proteins showed the highest protection from histopathology, weight loss, and viral loads in golden Syrian hamsters. Finally, Kim and co-workers [93] used *Mycobacterium paragordonae* to develop a novel vector system (rMpg-RBD-7) expressing SARS-CoV-2 RBD, and this construct showed promising results in mice.

It is interesting to notice that no efforts were made to use bacteria typically found in the airways, which would naturally present a longer colonization span and lead to a potentially stronger and longer-lasting immunization profile. Moreover, no clinical trials have been performed to date. Therefore, further efforts in the field should be made, and other species should be tested as vector carriers for fragments of SARS-CoV-2 with possible immunogenic activity.

## 6. Conclusions

A clearer understanding of more intricate host–bacterial and interbacterial association networks that allow mutualistic, commensal, or (direct or indirect) antagonism in several distinct niches such as that of the upper respiratory tract allows for better design of bacterial mucosal vector vaccines. In the process of determining the main entry sites and targets for pathogenic infection, resident microbes are deemed necessary in host protection and immune homeostasis [112]. Hence, future attempts should focus on the resident microbiome data and mutualism of bacterial society to define targets for new mucosal vaccine vector development. In this regard, the focus should be directed to finding the dominant species, observing which species present in the community are pathogenic, and understanding their ecology regarding the colonization span (adhesion, growth rate, etc.).

Particular attention should be paid to resident commensal species with a protective function. The prophylactic effects of commensal probiotics and their byproducts help to maintain gut commensal microbiota and prevent invasion by pathogens. Potential probiotics have direct antiviral mechanisms, such as the secretion of S-layer proteins, bacteriocins, and an increase of zinc bioavailability. It also has other indirect antiviral effects, such as modulating the host immune system (IFN-γ, IgA, IL-12, NK cells, etc.) through the release of short-chain fatty acids and exopolysaccharides [115]. Therefore, resident commensal species present great potential in developing mucosal vaccines since they present several beneficial effects and ways to activate the immune system, maximizing the effect of such vaccines.

Finally, regarding SARS-CoV-2, efforts should be made to anticipate uncertainties and events that may be relevant to a long-term association with the virus, even though population-level immunity is within reach with the current vaccines available. It is important to keep in mind the possibility for SARS-CoV-2 to be an endemic virus, with potential for persistence through seasonal infection peaks, waning immunity protection, and exposure of susceptible individuals [116]. The current vaccine’s mechanism of action and limitations are still unclear, so continued vigilance, observation, preparation, and development of prophylactic policies, diagnostic, and therapeutic tools are necessary. In this aspect, we believe it is also important to continue investigating methods for fortifying our natural defenses, both mucosal and systemic. Novel approaches are now underway to design universal Sarbecovirus vaccines using structure-guided vaccine designs for SARS-CoV-2 variants, which will achieve broader B or T cell responses to end the current pandemic and prevent emerging new variants [91,117].

## Figures and Tables

**Table 1:** PAMPs and immune activation.

Molecule Fragment	Type Source	Immune Activation	Bacterial Vector Candidate	Cascade	Reference
Pyrophosphates	Bacterial isoprenoid synthesis	γδ T cells	*Bifidobacteriaceae*, *Bacillaceae*, and *E. coli* improve γδ T cells [57]	DC maturation; Neutrofil recruitment; Increase in tumour necrosis factor-α and interferon-γ	[31,40,57]
Acyl-homoserine lactones (AHL)-based QS	Quorum-sensing (QC) molecules	TLR4	Gram-negatives such as *Hafnia alvei*, *Edwardsiella tarda*, and *Ralstonia* sp.; *E. coli*, *Enterobacter*, and *Klebsiella* [58]	Recruitment of polymorphonuclear neutrophils; Production of interleukins that are dependent on the type of QS protein	[41,42,43,44,58]
Oligopeptide (AIP)-based QC	Quorum-sensing (QC) molecules	TLR4	Gram-positives such as *Staphylococcus aureus*, *Enterococcus faecalis*, *Streptococcus* *pneumoniae*, *Bacillus thuringiensis*, and *Lactobacillus* spp. [59]	Recruitment of polymorphonuclear neutrophils; Production of interleukins that are dependent on the type of QS protein	[41,42,43,44,59]
4, 5- dihydroxy-2, 3-pentanedione (DPD, AI-2)-based QS	Quorum-sensing (QC) molecules	TLR4	*Bacteroides vulgatus*, *Clostridium proteoclasticum*, *E. coli*, *Eubacterium rectale*, *Lachnospira multipara*, *Pseudobutyrivibrio ruminis*, *Roseburia intestinalis*, *Ruminococcus albus and Ruminococcus flavefaciens*, *Lactococcus lactis* [60]	Recruitment of polymorphonuclear neutrophils; Production of interleukins that are dependent on the type of QS protein	[41,42,43,44,60]
Quinolone-based QS	Quorum-sensing (QC) molecules	TLR4	*Pseudomonas aeruginosa* and related bacteria [61]	Recruitment of polymorphonuclear neutrophils; Production of interleukins that are dependent on the type of QS protein	[41,42,43,46,61]
cyclic-di-GMP	Cyclic dinucleotides	STING, DDX41	*Faecalibacterium prausnitzii*, *Eubacterium rectale*, *Mitsuokella multacida*, and commensal *E. coli* [62,63]	STING-TBK1-IRF3 pathway; DDX41; RECON; type I interferons and cytokines	[47,54,62,63]
cyclic-di-AMP	Cyclic dinucleotides	STING, DDX41, RECON	Biofilm-forming such as *Streptococcus sp.* and commensal *Escherichia coli* [64]	STING-TBK1-IRF3 pathway; DDX41; RECON; NF-kB, type I interferons, and cytokines	[54,55,64,65]
Lipopolysaccharide (LPS)	Component of cell wall	TLR4	Commensal Gram-negatives such as *E. coli*, *Hafnia alvei*, *P. aeruginosa*, *Morganella morganii*, *Pseudomonas putida*, *Citrobacter koseri*, and *Klebsiella* *pneumoniae* [66,67]	Induce nuclear factor-κB (NF-κB); tumour necrosis factor-α (TNF-α); interleukin (IL)–12	[51,66,67]
Flagellin residues 89–96	Bacterial flagellin	TLR5	Commensal Firmicutes such as *Roseburia* (R. *inulivorans*, *R. intestinalis*), *Eubacterium sp.*, and *Clostridium* (*Clostridium scindens*, *C. ramosum*, *C. bolteae*, *C. bartletti*), and commensal Proteobacteria such as *Providencia stuartii*, *Citrobacter amalonaticus*, and *S*. *Typhimurium* [68]	MyD88-dependent; Activates NFkB and MAPK pathways	[53,68]

**Table 2:** Bacteria used as mucosal vectors.

General	Bacterial Species Vector	Expressed Antigen/Immunogen	Reference
*Salmonella* spp.	*Salmonella enterica* serovar Typhimurium (live-attenuated)	*Mycobacterium tuberculosis* early secreted antigenic target 6-kDa (ESAT-6) protein and culture filtrate protein 10 (CFP-10) antigens	[76]
	*Salmonella enterica* serovar Typhi (live-attenuated)	*Pseudomonas aeruginosa* highly conserved outer membrane proteins OprF and OprI	[77]
	*Salmonella enterica* serovar Typhi (live-attenuated)	*Streptococcus pneumoniae* surface protein antigen PspA	[78]
	*Salmonella enterica* serovar Typhimurium (live-attenuated)	Cytolysin A (ClyA)-Spike protein of SARS-CoV1 (S1E)	[79]
*Listeria* spp.	*Listeria monocytogenes* (live-attenuated)	*Mycobacterium tuberculosis* 30 kDa major secretory protein (r30/antigen 85B (Ag85B))	[80]
*Bacillus* spp.	*Bacillus subtilis*	*Bacillus anthracis* protective antigen	[81]
	*Bacillus subtilis*	*Mycobacterium tuberculosis*, *Antigen MPT64*	[82]
	*Bacillus subtilis*	*Mycobacterium tuberculosis*, *Secretory antigens Ag85B* and *CFP10*	[83]
*Lactococcus lactis*	*Lactococcus lactis*	SARS-CoV N protein	[84]
*Lactobacillus* spp.	*Lactobacillus*	*Streptococcus pneumoniae*, PsaA	[85]
	*Lactobacillus casei*	CTA1-conjugated Influenza sM2 protein	[86]
	*Lactobacillus casei*	CTA1-conjugated Influenza sM2 and HA1	[87]
	*Lactobacillus casei*	PgsA-Spike (S) protein segments SA (residues 2 to 114) and SB (residues 264 to 596) of SARS-CoV	[88]
	*Lactobacillus plantarum*	*Mycobacterium tuberculosis*, Fusion antigen AgE6	[89]
	*Lactobacillus plantarum*	SARS-CoV-2 Spike protein (whole protein)	[90]
	*Lactobacillus plantarum*	Receptor-binding domain (RBD) of the SARS-CoV-2 Spike protein	[91]
*Novel vector host*	*Francisella tularensis* subsp. *holarctica* (single live multi-deletional attenuated)	SARS-CoV-2 Spike, envelope, membrane, and nucleocapsid proteins	[92]
	*Mycobacterium paragordonae*	Receptor-binding domain (RBD) of the SARS-CoV-2 Spike protein	[93]

## Data Availability

Not applicable.
